# Night Eating Syndrome in Patients With Obesity and Binge Eating Disorder: A Systematic Review

**DOI:** 10.3389/fpsyg.2021.766827

**Published:** 2022-01-05

**Authors:** Jasmine Kaur, An Binh Dang, Jasmine Gan, Zhen An, Isabel Krug

**Affiliations:** Melbourne School of Psychological Sciences, The University of Melbourne, Melbourne, VIC, Australia

**Keywords:** night eating syndrome, binge eating disorder, eating disorder pathology, systematic review, obesity, bariatric surgery

## Abstract

Night eating syndrome (NES) is currently classified as an Other Specified Feeding or Eating Disorder (OSFED) under the Diagnostic Statistical Manual−5 (DSM-5). This systematic review aims to consolidate the studies that describe the sociodemographic, clinical and psychological features of NES in a population of patients with eating disorders (ED), obesity, or those undergoing bariatric surgery, and were published after the publication of the DSM-5. A further aim was to compare, where possible, NES with BED on the aforementioned variables. Lastly, we aimed to appraise the quality of the studies being included in the review. We conducted a systematic search on three databases (MEDLINE, PubMed and Embase) which resulted in the selection of 22 studies for the review. We included the articles that studied patients with NES and their sociodemographic, clinical and psychological features in a clinical (i.e., ED, obese or bariatric surgery) population, through a quantitative study design. Articles were excluded if the NES patients included in the study had a comorbid psychological disorder, and/or the sample was collected from a university/non-clinical population, and/or the study design was qualitative, and/or NES features were compared with any other disorder, except BED. Our study found that no conclusions about the link between any sociodemographic feature (such as, age, gender, income, etc.) and an NES diagnosis could be made. Further, NES patients presented with elevated ED pathology (including emotional eating and loss of control eating) and higher occurrence of depressive symptoms than controls. Contrary to the literature suggesting that NES and Binge Eating Disorder (BED; an ED subtype which is also comorbid with obesity) patients often report overlapping features, questioning the validity of NES as an ED diagnosis, we found that BED can be differentiated from NES by the higher occurrence of emotional eating, body related concerns and abnormal eating episodes. The review also suggested an overlap between NES and Sleep-Related Eating Disorder. We recommend that it is essential to study NES as an independent disorder to further develop its diagnostic criteria and treatment options, thereby, increasing the quality of life of the patients suffering from this syndrome.

## Introduction

The switch from the 4th edition of the Diagnostic Statistical Manual (DSM-IV-TR; American Psychiatric Association, 2000) to the 5th edition (DSM-5; American Psychiatric Association, 2013) saw significant changes to the diagnoses of eating disorders (ED). Changes ranged from the characterization of Binge Eating Disorder (BED) as an independent ED to the introduction of new ED categories, for example, Other Specified Feeding or Eating Disorder (OSFED) and Unspecified Feeding or Eating Disorder (UFED; Mancuso et al., 2015). The characterization of Night Eating Syndrome (NES) was also affected by this transition to the DSM-5 (American Psychiatric Association, 2013). In DSM IV-TR (American Psychiatric Association, 2000), NES was present under the category of Eating Disorder Not Otherwise Specified (EDNOS) but has now been characterized as an OSFED, after EDNOS was dropped as a category of EDs in the DSM-5 (^*^Roer et al., 2014; use of ^*^ denotes that the cited paper was selected for data extraction in this systematic review).

NES, which was first described in 1955, has only recently received the attention it requires to develop a comprehensive diagnostic criterion. The DSM-IV-TR (American Psychiatric Association, 2000) criteria for NES were quite simplistic and included morning anorexia, evening hyperphagia, and insomnia. Allison et al.'s (2010) efforts to improve the criteria for diagnosing NES, led to a fruitful change in the way symptoms of NES are now described in the DSM-5 (American Psychiatric Association, 2013). Their proposed criteria includes: (A) evening hyperphagia, defined as the consumption of 25% or more of total daily caloric intake after the evening meal, and/or two nocturnal ingestions on average per week; (B) awareness of one's eating behaviors and ingestions; (C) three of the following: (i) morning anorexia, (ii) the desire to eat between dinner and sleep, (iii) sleep onset insomnia, (iv) the belief that one needs to eat in order to fall asleep, (v) depressed or lowering of mood in the evening and night time; (D) distress or impaired functioning; (E) a period of 3 months or more under these conditions; and (F) these conditions are not secondary to any medical condition.

Unlike some other EDs, such as Anorexia Nervosa, NES is not dependent on an individual's Body Mass Index (BMI). NES can occur in people within the normal range of weight corresponding to their age and height, however, is most found and researched in individuals with obesity (Aronoff et al., 2001). This is important because NES has been found to be a risk factor for an earlier onset of obesity and is related to higher rates of depression and lower self-esteem (Striegel-Moore et al., 2009). Most patients undergoing bariatric surgery have a high BMI and suffer from obesity, hence, they are at risk for developing NES too. Overall, comorbidity of NES and obesity has been linked to a poorer prognosis for long-term weight loss and maintenance, especially in bariatric surgery patients (Vander Wal, 2012).

BED and NES are thought to be very similar disorders due to their higher rate of occurrence in patients with obesity and their strong links with depressive symptoms (Allison et al., 2007). However, Latzer and Tzischinsky's (2012a) work has outlined differences between BED and NES in terms of the amount and time of food intake. They argue that a key differentiating factor between NES and BED patients is the portion size of the food intake. While BED patients experience “Loss of Control” (LoC) over eating and consume uncharacteristically large meals, NES patients tend to retain their control and “snack” at atypical times, i.e., at night or after the evening meal (Stunkard and Allison, 2003).

Another key debate in the literature is around its classification as an ED (Shoar et al., 2019), which is especially controversial because of the overlap of NES symptoms with Sleep-Related Eating Disorder (SRED). Patients with NES and SRED both present with nightly binging, an increase in weight, and a disruption in sleep (Winkelman, 2006). Moreover, both are highly prevalent amongst women and are often comorbid with depression. It has been argued that these similarities in symptoms may indicate that the two disorders are the same but have previously been studied through two different lenses in the literature (Winkelman, 2006). The only key difference noted in the literature is the level of consciousness of the individual during the night eating episode. While NES patients are fully conscious and aware of their night binging episode(s), SRED patients have partial or no consciousness of the night eating episode (Winkelman, 2006). Supporting this claim, researchers such as Latzer and Tzischinsky (2012b) believe that NES should be considered an ED because of the presence of psychopathological features related to disordered eating (such as binge eating and emotional eating). However, the differences between these two disorders are still under-researched.

### The Current Review

Given that most of the literature relating to NES has been published in recent years—after the publication of the DSM-5 in 2013 (American Psychiatric Association, 2013)—and the research from before the publication of the DSM-5 is redundant according to the current definition of NES, only studies published after 2013 were included in the current review. When coupled with the above–mentioned gaps, inconsistencies and controversies in the literature, a synthesis of relevant studies assessing NES is both timely and warranted. Therefore, we aimed to: (a) summarize the known sociodemographic, clinical, and psychological features of NES in patients with ED, obesity and/or those undergoing bariatric surgery; (b) compare, where possible, NES with BED on these variables; and (c) undertake a quality appraisal of all the studies included in the review.

Research has shown that a significant proportion of comorbidity between obesity and EDs (especially BED) exists. Accordingly, studies have shown that up to 5–15% (McCuen-Wurst et al., 2018) of patients with obesity presented with BED and/or NES, and this number climbed up to 50% (Succurro et al., 2015) in bariatric surgery patients. The current review, therefore, included studies undertaken with an ED population as well as individuals suffering from obesity, which itself includes individuals who may have undergone or are planning to undergo bariatric surgery.

This review is important because presently NES has varying definitions across different papers (Striegel-Moore et al., 2009). This is because of the dynamic understanding of its diagnostic criteria, symptoms, and associated features over time. The controversies around the overlap of its features with BED and SRED add to our lack of understanding of this disorder. Therefore, this review becomes essential in collating our knowledge about NES and its sociodemographic, clinical, and psychological features, while attempting wherever possible to differentiate it from BED, a closely related ED. Finally, a quality appraisal of the included studies will allow us to critically analyse and interpret the data extracted from these studies for the purpose of this review.

## Method

### Search Strategy

For this review, a systematic search was conducted on three international databases—MEDLINE, PubMed and Embase—to gather relevant literature about NES and how it differs from BED. The Preferred Reporting for Systematic Reviews and Meta-Analyses (PRISMA) guidelines (Moher et al., 2009) were followed to complete this systematic search. The review was also registered on PROSPERO: International Prospective Register for Systematic Reviews developed by University of York (ID: CRD42021233024; 2nd September 2021; https://www.crd.york.ac.uk/prospero/display_record.php?ID=CRD42021233024). Two searches were undertaken: the first where NES related keywords (“night eating syndrome” OR “night eating” OR “late night eating” OR “night eating disorder”) were input, and the second where NES related keywords were conjugated using “AND” with BED related terms (“binge eating disorder” OR “binge eating” OR “binge” OR “binging”) to obtain studies published after 2013 (i.e., after the publication of DSM-5), until the 25th of August, 2021. Filters were applied to limit the search to studies performed with human participants and reported in English. No unpublished data was included in this review. The complete search syntax is provided as Supplementary Material.

### Inclusion and Exclusion Criteria

Studies were judged for inclusion in the review based on the following criteria:

(a) the NES diagnosis was given when a person—(i) met the DSM-5 (American Psychiatric Association, 2013) criteria for NES, or (ii) received a score ≥ 25 on the Night Eating Questionnaire (NEQ) developed by Allison et al. (2008), or (iii) met the criteria set by the Night Eating Diagnostic Questionnaire (NEDQ; Gluck et al., 2001), or (iv) met the criteria set by the Night Eating Symptom and History Inventory (NESHI; an unpublished, semi-structured interview developed by Stunkard's team); and (b) the study should have measured at least one of the following features of NES patients—(i) sociodemographic (e.g., age, BMI, etc.), (ii) clinical, ED related and/or other comorbid psychopathology (e.g., loss of control eating, binging episodes, sleep features, etc.), (iii) personality factors, and (iv) treatment paradigms; and (c) if a paper studied a clinical population (limited to patients with ED/obesity/undergoing bariatric surgery); and (d) if a quantitative study design was employed (e.g., case-control studies using standardized questionnaires and/or experimental procedures).

Studies were excluded if:

(a) the paper studied NES in a university or non-clinical population; (b) NES patients had a comorbid psychological disorder such as depression or schizophrenia; (c) if the data collected was qualitative in nature (e.g., reviews or case reports); (d) compared NES to another medical or mental disorder, but not BED. University students were excluded as a population because of an existing prospective review registered on PROSPERO (ID: CRD42020161014; 28th April 2020; https://www.crd.york.ac.uk/prospero/display_record.php?ID=CRD42020161014). The clinical population was restricted to ED and obese/bariatric surgery patients to extract the most useful information particular to NES.

### Quality Appraisal

Quality of the studies included in the systematic review was evaluated using an adapted version of the framework created by Downs and Black (1998) for clinical trials. Rozenblat et al. (2017) adapted the following 13 criteria for the purpose of ED research:

1) Clear description of the hypothesis/aim/objectives; 2) Clear description of main outcomes in introduction/method; 3) Participant characteristics clearly described (as appropriate for ED research); 4) Clear description of main findings; 5) Characteristics of participants lost to follow-up described; 6) Exact probability values reported (or confidence intervals included); 7) Participants representative of population (including clinical, but not convenience samples); 8) Any “data-dredging” explicitly noted; 9) Appropriate statistical tests used; 10) Main outcome measures valid and reliable; 11) Participants in different groups (if case- control study) recruited contemporaneously; and 12) Adequate adjustment for (potential) confounding variables (e.g., BMI); 13) Sufficient power (to detect a GxE interaction). This review employed an adapted version of Rozenblat et al.'s (2017) criteria as criteria 8 and 13 stand irrelevant in the context of the present study. None of the studies explicitly noted “data-dredging” and no gene X environment interactions are relevant for this review.

Studies were evaluated by JK and AZ independently. All discrepancies were discussed by the two coders and resolved with IK, the supervisor of this project.

## Results

### Systematic Search

The first systematic search using NES-related keywords resulted in 1,297 studies across the three academic databases, which reduced to 687 studies after the duplicates were removed. Next, the second search using NES-related keywords AND BED-related keywords was conducted to find studies focusing on the overlap of the two disorders. This search yielded 577 studies, which reduced to 362 when duplicates were removed using EndNote. Further, the studies from the first and the second search were manually compared to find duplicates in the two searches. All 362 studies from the second search were already included in the 687 studies from the first search, therefore, the 687 studies from the first search were selected for the title and abstract screening. At this stage of the screening, 545 studies were excluded based on the inclusion and exclusion criteria. The screening was conducted by JK and AZ independently and the inter-rater reliability was 66.87%, while the discrepancies were resolved by discussing them with IK.

Of the remaining 142 studies, 61 were excluded because they utilized the NES criteria that were used before the publication of the DSM-5 (American Psychiatric Association, 2013). Another 24 studies were excluded because only abstracts were available for them, and one study could not be located for further screening. Therefore, for the next stage of full-text screening, only 56 studies were available. Further, 34 studies were excluded because: (i) 14 studies did not meet inclusion criteria for one or more of the outlined clinical populations (i.e., ED, obesity or bariatric patients); (ii) 12 studies did not have relevant outcomes according to the research questions; (iii) five studies had qualitative study designs; and (iv) three studies were not in English and could not be read in full.

**Figure 1:**
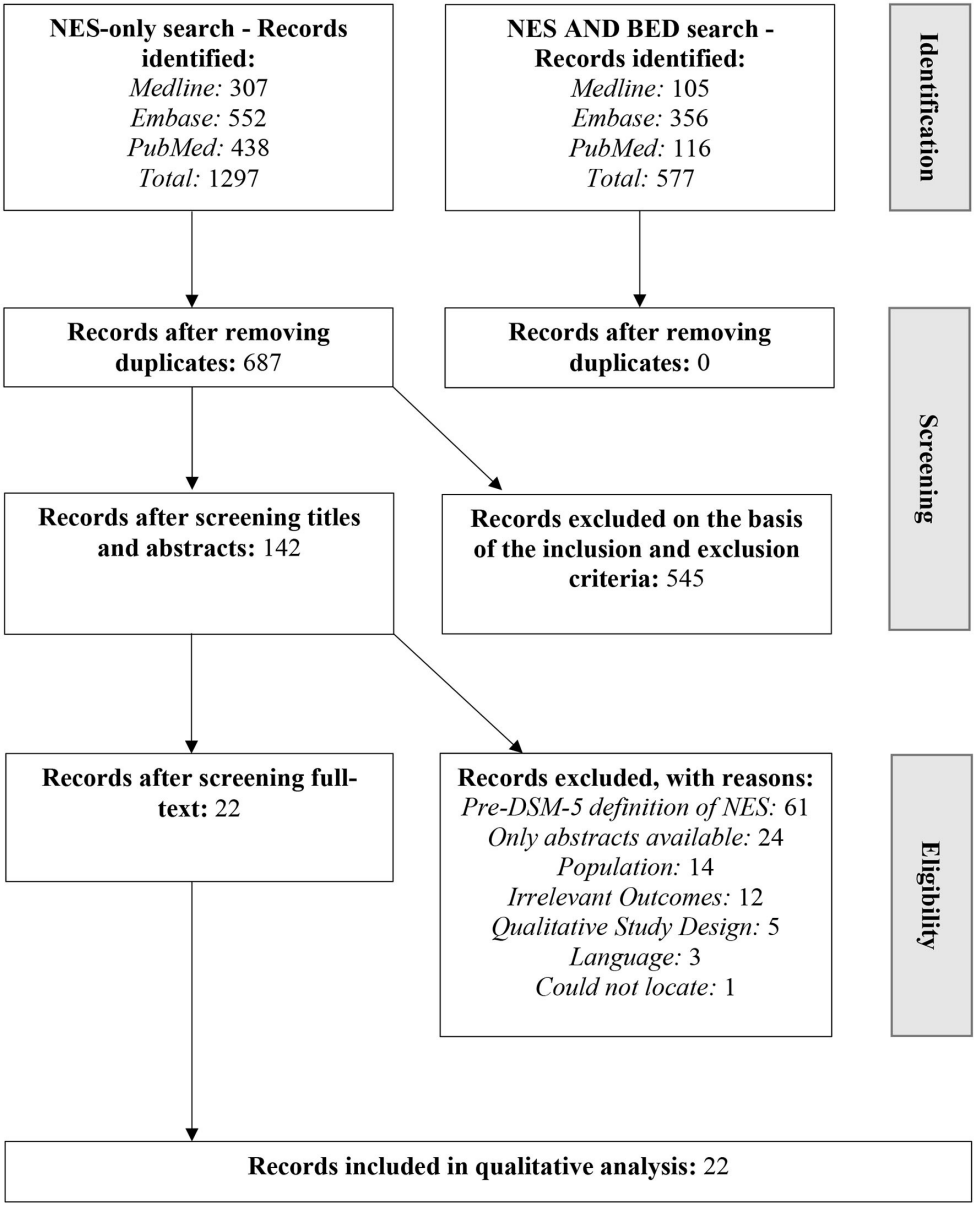
PRISMA flow chart for selection of studies for the systematic review.

The remaining 22 studies (^*^Cleator et al., 2013, 2014; ^*^Dalle Grave et al., 2013; ^*^Roer et al., 2014; ^*^Baldofski et al., 2015; ^*^McCune and Lundgren, 2015; ^*^Royal et al., 2015; ^*^Vander Wal et al., 2015; ^*^Vinai et al., 2015a,b; ^*^Dorflinger et al., 2017; ^*^Ferreira Pinto et al., 2017; ^*^Nasirzadeh et al., 2018; ^*^Zengin-Eroglu et al., 2018; ^*^Loddo et al., 2019; ^*^Tu et al., 2019; ^*^Zickgraf et al., 2019; ^*^Kara et al., 2020; ^*^Latzer et al., 2020a,b; ^*^Uncu et al., 2020; ^*^Ivezaj et al., 2021) were included in the systematic review for extraction of data (see Figure 1 for the overview of the study selection).

### Characteristics of Selected Studies

Table 1 summarizes the 19 studies that collected various anthropometric data and measured psychological outcomes for patients with NES. Table 2 summarizes the three studies that outline the similarities and differences found in NES and BED patients based on demographic and psychological outcomes. For all 22 studies, a total of 4,502 participants were recruited across various clinical populations, i.e., ED patients (including NES) (*n* = 947 across eight studies; ^*^McCune and Lundgren, 2015; ^*^Vander Wal et al., 2015; ^*^Vinai et al., 2015a,b; ^*^Loddo et al., 2019; ^*^Tu et al., 2019; ^*^Latzer et al., 2020a,b), patients with obesity (*n* = 1,616 participants across seven studies; ^*^Cleator et al., 2013, 2014; ^*^Dalle Grave et al., 2013; ^*^Roer et al., 2014; ^*^Dorflinger et al., 2017; ^*^Kara et al., 2020; ^*^Uncu et al., 2020), and those who have had bariatric surgery or are planning to undergo one (i.e., prebariatric surgery patients) (*n* = 1,939 participants across seven studies; ^*^Baldofski et al., 2015; ^*^Royal et al., 2015; ^*^Ferreira Pinto et al., 2017; ^*^Nasirzadeh et al., 2018; ^*^Zengin-Eroglu et al., 2018; ^*^Zickgraf et al., 2019; ^*^Ivezaj et al., 2021).

**Table 1:** Studies investigating NES in patients with eating disorder(s), obesity, and/or undergoing bariatric surgery.

**References**	**Sample attributes**	**Results: Demographic data**	**Outcomes (Measures)**	**Results: Outcomes**
1. Cleator et al. (2013)	Population: Patients with severe obesity *N* = 144 (68% female) (mean age of *M* = 44.6, *SD* = 12.1; mean BMI of *M* = 46.9 kg/m^2^, *SD* = 9.5) Country: United Kingdom	Prevalence: Individuals with NEQ scores ≥ 25, *n* = 20 (13.9%) Individuals with NEQ scores ≥ 30, *n* = 4 (2.8%)	NES (NEQ) Sleep Quality (PSQI) OSA (ESS)	Sleep Quality: Poor levels of sleep quality in patients with obesity Night eating is rare, but sleep quality highly negatively correlated with NEQ scores, even when controlling for ESS scores
2. Cleator et al. (2014)	Population: Patients with severe obesity *N* = 81 (mean age of *M* = 44.6, *SD* = 11.6; mean BMI of *M* = 50.0 kg/m^2^, *SD* = 10.7) Country: United Kingdom	Prevalence: Full-syndrome NES, *n* = 7; Partial NES, *n* = 24; NEB = Full-syndrome NES + Partial NES = 31; Non-NEB, *n* = 50 Age, gender, BMI: No significant difference Weight: NEB > non-NEB Employment: NEB patients less likely to be working	NES (NESHI, NEQ) Depression (BDI-IA)	Depression, anxiety: No significant difference between groups BDI-IA (greater depressed mood): NEB > non-NEB Sleep duration: No significant difference between groups; NEB group had greater within group sleep duration variability
3. Dalle Grave et al. (2013)	Population: Patients with obesity *N* = 586 (100% female) (mean age of *M* = 47.7, *SD* = 9.8; mean BMI of *M* = 38.2 kg/m^2^, *SD* = 6.8) Controls: *N* = 185 (100% female) (mean age of *M* = 47.2, *SD* = 10.7; mean BMI of *M* = 22.8 kg/m^2^, *SD* = 1.7) Country: Italy	Prevalence: NES (NEQ score ≥ 30): 18 (3.1%) participants with obesity, of which 17 had severe BED as well Age, BMI: No significant difference	NES (NEQ) Binge Eating (BES) Personality characteristics (TCI)	Higher scores on NEQ = Higher BMI BES scores: NES > non-NES TCI–Novelty seeking, Reward dependence, Persistence, Cooperativeness, Self-transcendence: No significant difference between NES and non-NES TCI–Harm avoidance: NES > non-NES TCI–Self-directedness: NES < non-NES
4. Dorflinger et al. (2017)	Population: Veterans with obesity *N* = 126 (90% male) (mean age of *M* = 61.6, *SD* = 8.5; mean BMI of *M* = 38.0 kg/m^2^, *SD* = 7.4) Country: United States of America	Prevalence: NES: 11% participants (83.3% screened positive for insomnia) Age, gender, BMI: No significant difference	Insomnia (ISI) NES (NEQ) Depression (PHQ-2) Post-Traumatic Stress Disorder (PC-PTSD) ED Pathology (EDE-Q, EOQ, MOVE!23, QEWP-R)	∙ Higher scores on NEQ = Higher BMI, Higher insomnia, Higher binge eating, Higher emotional eating, Higher eating disorder symptomology ∙ For veterans with NES: more likely to screen positive for depression and PTSD (when controlled for BMI)
5. Ferreira Pinto et al. (2017)	Population: Bariatric Surgery *N* = 60 (85% female) (mean age of *M* = 34.7, *SD* = 9.2; mean BMI of *M* = 46.04 kg/m^2^, *SD* = 7.52) Country: Brazil	NES patients before bariatric surgery vs. after surgery: 7 vs. 6	NES (NEQ) Depression (BDI-SF)	∙ Reduction in global NEQ scores observed after bariatric surgery (*P* = 0.05) ∙ Reduction in NES post-surgery was greater in patients with baseline depressive symptoms (*P* = 0.03)
6. Ivezaj et al. (2021)	Population: Post-bariatric surgery *N* = 131 (84.7% female) (mean age of *M* = 45.5, *SD* = 10.9; mean BMI of *M* = 37.6 kg/m^2^, *SD* = 7.1) Country: United States of America	Prevalence: NES: *n* = 17 (15.2%) exceeded the threshold for NES on NEQ; *n* = 10 (8.9%) exceeded the threshold for “strongly suggestive NES;” *n* = 7 (6.3%) exceeded the threshold for “suggestive NES” Age, BMI, BMI Change, Sex: No significant difference Race: Significantly greater night eating in non-white racial groups (67.9%) Total Weight Loss % (TWL%): Lower TWL% in regular night eaters	NES (NEQ) ED Pathology (EDE-Bariatric Surgery Version) Depression (BDI-II) Sleep Quality (PSQI)	Greater EDE over-evaluation, EDE dissatisfaction, BDI-II scores, PSQI scores, LoC eating frequency than subthreshold night eaters: NES, strongly suggestive NES, suggestive NES groups > group with no or little night eating behavior No significant differences for EDE restraint scores were observed
7. Kara et al. (2020)	Population: Patients with obesity *N* = 421 (83% female) Class I Obesity Group: *N* = 150 (mean age of *M* = 49.49, *SD* = 12.49; mean BMI of *M* = 32.40 kg/m^2^, *SD* = 1.34) Class II Obesity Group: *N* = 150 (mean age of *M* = 48.43, *SD* = 11.81; mean BMI of *M* = 37.36 kg/m^2^, *SD* = 1.34) Class III Obesity Group: *N* = 150 (mean age of *M* = 49.05, *SD* = 11.40; mean BMI of *M* = 44.97 kg/m^2^, *SD* = 4.63) Country: Turkey	Gender, Income level, Occupational status, Presence of children, Living-together subjects, Cigarette consumption: No significant difference between NES and non-NES obesity groups Age: NES Class II obesity < non-NES Class II obesity (only group with significant difference)	NES (NEQ)	Duration of Obesity, Dieting, Weight loss, Number of main meals per day, Number of snacks per day: No significant difference between the three obesity groups and between the NES and non-NES groups for each obesity class
8. Latzer et al. (2020a)	Population: Eating Disorder patients *N* = 68 (100% female) (mean BMI of *M* = 27.2 kg/m^2^, *SD* = 7) Night binging group (NES + BED or BN: *N* = 25 (44.4%) (mean age of *M* = 38.3, *SD* = 13.60; mean BMI of *M* = 29.50 kg/m^2^, *SD* = 6.7) BE (BED or BN): *N* = 34 (56.6%) (mean age of *M* = 25.7, *SD* = 5.8; mean BMI of *M* = 25.3 kg/m^2^, *SD* = 6.3) Country: Israel	Age: NES-BE > BE only Education level, Occupation, Smoking: No significant difference between NES-BE and BE only	Eating Pattern (Food Diary) Depression (BDI)	Higher depression, Higher binging episodes, Higher weekly binging frequency, Higher calorie consumption, Higher average fat consumption per day: NES-BE > BE only Higher average carbohydrate consumption per day: BE only > NES-BE
9. Latzer et al. (2020b)	Population: Eating Disorder patients *N* = 426 (100% female) (mean age of *M* = 35.56, *SD* = 12.91) Night binging group (NES + BED or BN: *N* = 74 (mean age of *M* = 40.70, *SD* = 1.54; mean BMI of *M* = 30.71 kg/m^2^, *SD* = 0.8) BED: *N* = 44 (mean age of *M* = 34.49, *SD* = 1.75; mean BMI of *M* = 32.62 kg/m^2^, *SD* = 1.0) BN: *N* = 40 (mean age of *M* = 27.05, *SD* = 1.25; mean BMI of *M* = 23.96 kg/m^2^, *SD* = 0.76) Country: Israel	Age, BMI: BN < BED < NES-BE	Childhood Trauma (Childhood Trauma Questionnaire) Psychological state (BSI) Depression (BDI) Self-esteem (Rosenberg Self-esteem Scale) ED (EDI-2, SCOFF questionnaire) NES (NEQ)	Psychopathology, Childhood Trauma (EDI-2, BSI, BDI, Rosenberg Self-esteem scale): No significant difference between groups after adjusting for age except physical neglect Physical Neglect: NES-BE > BN and BED
10. Loddo et al. (2019)	Population: NES patients *N* = 20 (65% female) (mean age of *M* = 48, *SD* = 12; mean BMI of *M* = 30 kg/m^2^, *SD* = 9) Country: Italy	Evening Hyperphagia (EH): *N* = 10 (mean age of *M* = 56, *SD* = 5; mean BMI of *M* = 37 kg/m^2^, *SD* = 8) Nocturnal Ingestion (NI): *N* = 10 (mean age of *M* = 39, *SD* = 11; mean BMI of *M* = 24 kg/m^2^, *SD* = 3)	Sleep Quality and features (VPSG)	Sleep features (such as sleep time, sleep efficiency, sleep latency, sleep stage REM, REM latency): No significant difference between NI and EH subgroups Eating latency, Sleep latency after eating offset, Total eating episode duration: EH > NI subgroup
11. McCune and Lundgren (2015)	Population: NES patients *N* = 15 (mean age of *M* = 42.2, *SD* = 15.03; mean BMI of *M* = 29.4 kg/m^2^, *SD* = 7.4) Country: United States of America	Same as sample attributes	NES (NESS) Depression (BDI-II) Insomnia (ISI)	Night eating symptoms, insomnia, depressive symptoms all significantly reduce after Bright Light Therapy
12. Nasirzadeh et al. (2018)	Population: Bariatric surgery patients *N* = 844 (81.2% female) (mean age of *M* = 45; mean BMI of *M* = 48.6 kg/m^2^, *SD* = 7.8) Country: Canada	Same as sample attributes	NES (NEQ)	•NEQ scores reduce after all types of bariatric surgeries •NEQ scores rise significantly 3 years post-operation
13. Royal et al. (2015)	Population: Bariatric Surgery *N* = 226 (mean age of *M* = 43.8, *SD* = 10.9; mean BMI of *M* = 49.8 kg/m^2^, *SD* = 8.3) Country: United States of America	Same as sample attributes	ED Psychopathology (EDE-Q) NES (NEQ) Depression (PHQ-9) Alcohol use (AUDIT) Health related Quality of Life (SF-36)	Higher NEQ scores: Individuals with Loss of Control eating > individuals without Loss of Control eating NES criteria met on NEQ: Individuals with Loss of Control eating and individuals without Loss of Control eating did not meet criteria
14. Tu et al. (2019)	Population: Eating Disorder Patients *N* = 167 AN: *N* = 29 (mean age of *M* = 24.6, *SD* = 7.9; mean BMI of *M* = 15.4 kg/m^2^, *SD* = 2.5) BN: *N* = 63 (mean age of *M* = 27.0, *SD* = 7.0; mean BMI of *M* = 21.6 kg/m^2^, *SD* = 3.1) BED: *N* = 29 (mean age of *M* = 33.7, *SD* = 8.4; mean BMI of *M* = 28.4 kg/m^2^, *SD* = 5.4) Country: Taiwan	Prevalence: NES only, *n* = 15 (mean age of *M* = 27.7, *SD* = 8.0; mean BMI of *M* = 23.0 kg/m^2^, *SD* = 7.1) NES in patients with AN, *n* = 3 (10.3%) NES in patients with BN, *n* = 22 (34.9%) NES in patients with BED, *n* = 15 (51.7%)	NES (NESHI, NEQ, MES) ED (EDE-Q) Depression (BDI) Sleep Quality (PQSI)	∙ All NES-only patients suffered from EH ∙ 53.3% had more than two occurrences of nocturnal eating/week with a mean of *M* = 4.1, *SD* = 4.9 occurrences ∙ Scores on NEQ, EDE-Q, BDI, PQSI: NES only > Control
15. Uncu et al. (2020)	Population: Obesity patients *N* = 177 Obese group: *N* = 90 (mean age of *M* = 33.4, *SD* = 6.7; mean BMI of *M* = 43.9 kg/m^2^, *SD* = 4.1) Non-obese group: *N* = 87 (mean age of *M* = 34.8, *SD* = 9.0; mean BMI of *M* = 25.9 kg/m^2^, *SD* = 3.1) Country: Turkey	Same as sample attributes	NES (NEQ) Impulsivity (BIS)	∙ NES was more frequent in the obese group with higher scores on NEQ (*p* < 0.001) ∙ NEQ and BIS correlated significantly in the positive direction (*p* < 0.005)
16. Vander Wal et al. (2015)	Population: NES patients *N* = 44 (70% female) (mean age of *M* = 46.07, *SD* = 12.67; mean BMI of *M* = 31.31 kg/m^2^, *SD* = 5.90) Country: United States of America	Same as sample attributes	NES (NEDQ, NEQ) Depression (BDI-II) Anxiety (BAI) Stress (PSS) Sleep (Sleep diary, accelerometer)	∙ Post-treatment reduction in NES, depression, anxiety, and stress in all three intervention groups (i.e., psychoeducation, practicing progressive muscle relaxation (PMR) therapy, and exercising while practicing PMR) ∙ Greatest reduction in food eaten after evening meal in the PMR group (−30.54%), being significantly different from the education group
17. Vinai et al. (2015a)	Population: NES patients *N* = 54 Night eaters: *N* = 24 (mean age of *M* = 44.30, *SD* = 10.90; mean BMI of *M* = 26.30 kg/m^2^, *SD* = 3.88) Controls: *N* = 30 (mean age of *M* = 45.80, *SD* = 11.27; mean BMI of *M* = 23.10 kg/m^2^, *SD* = 3.63) Country: Italy	NES patients have a higher BMI (*p* = 0.003)	ED psychopathology (EDI-2) BN (BITE) Personality (TCI) Impulsivity (BIS) Sleep features (VPSG)	EDI-2 (drive for thinness, interceptive awareness, impulse regulation): NES > controls BITE (symptom score only): NES > controls TCI (harm avoidance): NES > controls
18. Vinai et al. (2015b)	Population: NES patients and insomniacs *N* = 153 (66% female) NES: *N* = 34 (mean age of *M* = 55, *SD* = 15.4; mean BMI of *M* = 29.4 kg/m^2^, *SD* = 4.9) Insomniacs: *N* = 47 (mean age of *M* = 57.2, *SD* = 10.0; mean BMI of *M* = 27.1 kg/m^2^, *SD* = 4.2) Controls: *N* = 72 (mean age of *M* = 50.5, *SD* = 17.7; mean BMI of *M* = 24.4 kg/m^2^, *SD* = 3.3) Country: Italy	Age, sex: No significant differences between groups	Alexithymia (TAS) NES (NEQ)	TAS total score: NES < Insomniacs EOT scale of TAS: NES < Comparison < Insomniacs
19. Zengin-Eroglu et al. (2018)	Population: Bariatric Surgery Participants *N* = 108 (80.6% female) (mean age of *M* = 37.50, *SD* = 9.10; mean BMI of men *M* = 45.77 kg/m^2^, *SD* = 6.16; mean BMI of women *M* = 45.45 kg/m^2^, *SD* = 4.70) Country: Turkey	Prevalence: BED, *n* = 12 (11.1%), NES, *n* = 6 (5.6%) Both BED and NES, *n* = 7 (6.5%) BMI, Education: did not differ across groups	Depression (BDI) Eating behavior (EAT)	No data for NES patients specifically

*NES, Night Eating Syndrome; BED, Binge Eating Disorder; ED, Eating Disorder; BN, Bulimia Nervosa; BE, Binge Eating; AN, Anorexia Nervosa; BMI, Body mass index; EDE-Q, Eating Disorder Examination-Questionnaire; NEB, Night Eating Behavior; NESHI, Night Eating Symptom and History Inventory; NEQ, Night Eating Questionnaire; BDI-IA, Beck Depression Inventory; BES, Binge Eating Scale; TCI, Temperament and Character Inventory; ISI, Insomnia Severity Index; PHQ, Patient Health Questionnaire; PC-PTSD, Primary Care PTSD Screen; EOQ, Emotional Overeating Questionnaire; QEWP-R, Questionnaire for Eating and Weight Patterns; BDI-SF, Beck Depression Inventory-Short Form; BSI, Brief Symptom Inventory; VPSG, Video-polysomnography; TAS, Toronto Alexithymia Scale; EOT, Externally-Oriented Thinking subscale; AUDIT, Alcohol use disorder identification test; SF-36, Short Form (36) Health Questionnaire; MES, Morningness/Eveningness Scale; PQSI, Pittsburgh Sleep Quality Index; BIS, Barratt's Impulsiveness Scale; NEDQ, Night Eating Diagnostic Questionnaire; BAI, Beck Anxiety Inventory; PSS, Perceived Stress Scale; EDI, Eating Disorder Inventory; BITE, Bulimic Investigatory Test-Edinburgh; EAT, Eating Attitudes Test; NESS, Night Eating Symptom Scale; OSA, Obstructive Sleep Apnoea; ESS, Epworth Sleepiness Score*.

**Table 2:** Studies investigating the differences and similarities between NES and BED in patients with eating disorder(s), obesity, and/or undergoing bariatric surgery.

**References**	**Sample attributes**	**Results: Demographic data**	**Outcomes (Measures)**	**Results: Outcomes**
20. Baldofski et al. (2015)	Population: Pre-bariatric surgery patients *N* = 233 (68% female) (mean age of *M* = 45.35, *SD* = 10.37; mean BMI of *M* = 48.72 kg/m^2^, *SD* = 7.64) Country: Germany	Prevalence: Full-syndrome NES, *n* = 19 (8.2%) patients; Sub-syndromal NES, *n* = 16 patients (6.9%) patients. Full Syndrome BED, *n* = 10 (4.3%) patients; Low frequency/limited duration BED, *n* = 12 (5.2%) patients; Sub-syndromal BED, *n* = 8 (3.4%) patients. Comorbid BED and NES, *n* = 9 (3.9%) patients.	Eating Disorder Psychopathology (EDE, EDE-Q, DEBQ-EE, EAH, YFAS)	Number of objective binge eating episodes: BED only > NES only > Non-ED Number of subjective binge eating episodes: NES only = BED only > non-ED Number of objective over eating episodes: NES only > non-ED = BED only Eating concern, Weight concern, Shape concern, and Global eating disorder psychopathology: BED only > non-ED = NES only Emotional eating, Eating without hunger, Number of food addiction symptoms: BED only = NES only > non-ED
21. Roer et al. (2014)	Population: Patients with obesity *N* = 81 (75% female) (mean age of *M* = 45.6, *SD* = 11.0; mean BMI of *M* = 38.0 kg/m^2^, *SD* = 8.5) Country: United States of America	Prevalence: BE only, *n* = 20 NES only, *n* = 9 BED and NES, *n* = 12 Controls, *n* = 35	Emotional Eating (EMAQ) BED (QEWP-R) NES (NEDQ)	Combined eating in response to negative emotions and situations: BED only > NES only, BD only > Controls, BED+NES > NES only Eating in response to negative situations: Controls > NES only, BED only > NES only, BED+NES > NES only Eating in response to negative emotions: BED only > Controls, BED only > NES only
22. Zickgraf et al. (2019)	Population: Pre-bariatric surgery patients *N* = 337 (mean age of *M* = 41.09, *SD* = 11.84; mean BMI of *M* = 48.33 kg/m^2^, *SD* = 7.56) Country: United States of America	Age, gender: No difference between food secure and food insecure groups Educational attainment: Food secure > Marginally food insecure > Food insecure PoC Ethnicity: Food secure < Food insecure	FIS (United States Department of Agriculture Adult Food Security Survey Module) Depression (BDI) BED (BES) NES (NEQ)	∙ FIS predicts NEQ scores more strongly than it predicts BED ∙ NEQ score prediction is partially mediated by depressive symptoms, predictability present directly and indirectly ∙ BED score prediction is fully mediated by depressive symptoms, predictability present only indirectly through BDI scores

*NES, Night Eating Syndrome; BED, Binge Eating Disorder; ED, Eating Disorder; BN, Bulimia Nervosa; BE, Binge Eating; AN, Anorexia Nervosa; BMI, Body mass index; EDE, Eating Disorder Examination; EDE-Q, Eating Disorder Examination-Questionnaire; DEBQ-EE, Dutch Eating Behavior Questionnaire—Emotional Eating; EAH, Eating in the Absence of Hunger Questionnaire; YFAS, Yale Food Addiction Scale; NEB, Night Eating Behavior; NEQ, Night Eating Questionnaire; BDI, Beck Depression Inventory; BES, Binge Eating Scale; QEWP-R, Questionnaire for Eating and Weight Patterns; EMAQ, Emotional Appetite Questionnaire; FIS, Food Insecurity Status; NEDQ, Night Eating Diagnostic Questionnaire*.

The 22 studies included in the review have been conducted across the globe—United States of America (*N* = 7; ^*^Roer et al., 2014; ^*^McCune and Lundgren, 2015; ^*^Royal et al., 2015; ^*^Vander Wal et al., 2015; ^*^Dorflinger et al., 2017; ^*^Zickgraf et al., 2019; ^*^Ivezaj et al., 2021), Italy (*N* = 4; ^*^Dalle Grave et al., 2013; ^*^Vinai et al., 2015a,b; ^*^Loddo et al., 2019), Turkey (*N* = 3; ^*^Zengin-Eroglu et al., 2018; ^*^Kara et al., 2020; ^*^Uncu et al., 2020), Israel (*N* = 2; ^*^Latzer et al., 2020a,b), United Kingdom (N = 2; ^*^Cleator et al., 2013, 2014), others (*N* = 4; ^*^Baldofski et al., 2015; ^*^Ferreira Pinto et al., 2017; ^*^Nasirzadeh et al., 2018; ^*^Tu et al., 2019).

Most of the samples in the included studies were predominantly female—ranging from 65 to 100% of the entire sample. The mean age of the participants ranged from 25 to 39 years for patients with EDs, 42 to 55 years for patients with NES, 33 to 62 years for patients with obesity, and 35 to 45 years for bariatric surgery and pre-bariatric surgery patients. The mean BMI range for each of the population sub-groups varied across studies-−15.40–32.62 kg/m^2^ for patients with ED, 26.30–31.31 kg/m^2^ for patients with NES, 32.40–50.00 kg/m^2^ for patients with obesity, and 45.45–49.80 kg/m^2^ for bariatric and pre-bariatric surgery patients.

Most of the selected studies (*N* = 19) employed questionnaires to measure the various outcomes of interest (such as, eating disorder pathology, loss of control eating, correlation with depression and anxiety, etc.); two of those (^*^Dalle Grave et al., 2013; ^*^Cleator et al., 2014) coupled the questionnaires with observational data collection methods (e.g., interviews). Ten studies (^*^Dalle Grave et al., 2013; ^*^Cleator et al., 2014; ^*^Roer et al., 2014; ^*^Baldofski et al., 2015; ^*^Royal et al., 2015; ^*^Vinai et al., 2015a,b; ^*^Tu et al., 2019; ^*^Kara et al., 2020; ^*^Uncu et al., 2020) employed a case-control design and two studies (^*^Baldofski et al., 2015; ^*^McCune and Lundgren, 2015) used a purely experimental design to test potential treatments for NES.

### Outcome Measures

A range of instruments were employed to identify the night eating symptoms in the included studies. The Night Eating Questionnaire (NEQ; developed by Allison et al., 2008; employed in *n* = 14 studies—^*^Cleator et al., 2013; ^*^Cleator et al., 2014; ^*^Dalle Grave et al., 2013; ^*^Royal et al., 2015; ^*^Vinai et al., 2015b; ^*^Dorflinger et al., 2017; ^*^Ferreira Pinto et al., 2017; ^*^Nasirzadeh et al., 2018; ^*^Tu et al., 2019; ^*^Zickgraf et al., 2019; ^*^Kara et al., 2020; ^*^Latzer et al., 2020b; ^*^Uncu et al., 2020; ^*^Ivezaj et al., 2021) was the most commonly used self-report measure. The NEQ is followed by the Night Eating Symptom and History Inventory (NESHI; employed in *n* = 3 studies–^*^Cleator et al., 2014; ^*^Vander Wal et al., 2015; ^*^Tu et al., 2019) and the Night Eating Diagnostic Questionnaire (NEDQ; developed by Gluck et al., 2001; employed in *n* = 2 studies–^*^Roer et al., 2014; ^*^Vander Wal et al., 2015) in the frequency of their use. Finally, the Night Eating Symptom Scale (NESS; developed by O'Reardon et al., 2006) was used in only one study (^*^McCune and Lundgren, 2015).

General ED symptoms were assessed with the Eating Disorder Examination-Questionnaire (EDE-Q; developed by Fairburn and Beglin, 1994; employed in *n* = 5 studies—^*^Baldofski et al., 2015; ^*^Royal et al., 2015; ^*^Dorflinger et al., 2017; ^*^Tu et al., 2019; ^*^Ivezaj et al., 2021), the Eating Disorder Inventory (EDI-2; developed by Garner et al., 1983; employed in *n* = 2 studies—^*^Vinai et al., 2015a; ^*^Latzer et al., 2020b) and the Questionnaire for Eating and Weight Patterns (QEWP-R; developed by Yanovski, 1993; employed in *n* = 1 studies—^*^Dorflinger et al., 2017), among other less frequently used measures (e.g., the Dutch Eating Behavior Questionnaire). Specifically, for the measurement of BED, studies employed the Binge Eating Scale (BES; developed by Gormally et al., 1982; employed in *n* = 2 studies—^*^Dalle Grave et al., 2013; ^*^Zickgraf et al., 2019) or the QEWP-R (employed in *n* = 1—^*^Roer et al., 2014).

Fifteen studies (^*^Dalle Grave et al., 2013; ^*^Cleator et al., 2014; ^*^McCune and Lundgren, 2015; ^*^Royal et al., 2015; ^*^Vander Wal et al., 2015; ^*^Vinai et al., 2015a; ^*^Dorflinger et al., 2017; ^*^Ferreira Pinto et al., 2017; Zengin-Eroglu et al., 2018; ^*^Tu et al., 2019; ^*^Zickgraf et al., 2019; ^*^Latzer et al., 2020a,b; ^*^Uncu et al., 2020; ^*^Ivezaj et al., 2021) assessed associated psychopathology using a range of measures, such as depression (e.g., Beck Depression Inventory; BDI; developed by Beck et al., 1961; employed in *n* = 10 studies), insomnia (e.g., Insomnia Severity Index; ISI; developed by Morin, 1993; employed in *n* = 2 studies), personality (e.g., Temperament and Character Inventory; TCI; developed by Cloninger et al., 1994; employed in *n* = 2 studies), impulsivity (e.g., Barratt's Impulsiveness Scale; BIS; developed by Barratt, 1965; employed in *n* = 2 studies), and alexithymia (i.e., difficulty in identifying and recognizing different inner emotional states; e.g., Toronto Alexithymia Scale; TAS; developed by Bagby et al., 1994; employed in *n* = 1 study). Food diaries (e.g., in ^*^Latzer et al., 2020a) and video-polysomnography (e.g., in ^*^Loddo et al., 2019) were also used by some studies to collect in-depth information about patient eating and sleep patterns respectively.

### Sociodemographic Features and Clinical Presentation

In this review, individuals with EDs, obesity and/or bariatric surgery patients were considered. In these clinical populations, we found that the overall prevalence of NES ranged from 2.8 to 8.2% where a NES diagnosis was endorsed upon receiving a score of >30 on the NEQ (^*^Cleator et al., 2013; ^*^Dalle Grave et al., 2013; ^*^Baldofski et al., 2015). However, with some leniency (i.e., NES being endorsed when a score of ≥ 25 was received on the NEQ), the prevalence increases to 6.9–15.2% (^*^Cleator et al., 2013; ^*^Baldofski et al., 2015; ^*^Dorflinger et al., 2017; ^*^Ivezaj et al., 2021). NES was found to be highly comorbid with other EDs: 51.7% NES patients had comorbid BED and 34.9% had comorbid Bulimia Nervosa (^*^Tu et al., 2019).

Multiple studies (*n* = 5; ^*^Dalle Grave et al., 2013; ^*^Cleator et al., 2014; ^*^Vinai et al., 2015b; ^*^Dorflinger et al., 2017; ^*^Ivezaj et al., 2021) provide evidence that age is not a risk factor for NES. However, ^*^Latzer et al. (2020a,b) have provided evidence that more older people get diagnosed with NES, while ^*^Kara et al. (2020) have argued otherwise stating that younger people with obesity are more likely to have NES. Similarly, BMI was found to not correlate with the diagnosis of NES by multiple studies (*n* = 5; ^*^Dalle Grave et al., 2013; ^*^Cleator et al., 2014; ^*^Dorflinger et al., 2017; ^*^Zengin-Eroglu et al., 2018; ^*^Ivezaj et al., 2021). However, contradictory findings were observed by ^*^Latzer et al. (2020b) and ^*^Vinai et al. (2015a), revealing a positive significant correlation between BMI and NES.

There is a consensus among the studies that other socio-demographic factors, such as gender (^*^Dalle Grave et al., 2013; ^*^Cleator et al., 2014; ^*^Vinai et al., 2015b; ^*^Dorflinger et al., 2017; ^*^Ivezaj et al., 2021), education level (^*^Zengin-Eroglu et al., 2018; ^*^Latzer et al., 2020a), income, having children, living together with a romantic partner, and smoking levels, were not different in NES individuals compared to controls (^*^Cleator et al., 2014; ^*^Kara et al., 2020). Few studies found lower rates of employment in the NES sample compared to a control group (^*^Cleator et al., 2014; ^*^Latzer et al., 2020a). Additionally, ^*^Ivezaj et al.'s (2021) recent study found greater levels of NES in non-white populations.

In terms of the clinical presentation of NES, most studies (*n* = 5; ^*^Cleator et al., 2013, 2014; ^*^Dalle Grave et al., 2013; ^*^Roer et al., 2014; ^*^Ivezaj et al., 2021) considered that a valid diagnosis of NES should be received when an individual scored more than 30 on the NEQ. Multiple studies (*n* = 4; ^*^Cleator et al., 2013, 2014; ^*^Roer et al., 2014; ^*^Ivezaj et al., 2021) differentiated between groups who scored more than 30 or more than 25 on the said scale. Astoundingly, in the studies by ^*^Roer et al. (2014) and ^*^Zengin-Eroglu et al. (2018), the prevalence of individuals with comorbid NES and BED conditions was found to be higher than the prevalence of individuals with only NES (14.8% with BED and NES both as compared to 11.1% with NES only; 6.5% with BED and NES both as compared to 5.6% with NES only, respectively).

### Eating Disorder Symptoms, General Psychopathology (Including Sleep), and Personality

The review focuses on synthesizing the psychopathological symptoms found in NES patients, including ED symptoms, general psychopathology and sleep pathology. Associations with certain personality traits are also briefly discussed.

In terms of ED symptoms, most studies found that NES correlated with a higher general ED pathology when compared to individuals with other EDs (e.g., BED and Bulimia Nervosa; ^*^Tu et al., 2019; ^*^Latzer et al., 2020a,b). Patients with NES were found to engage in emotional eating (^*^Dorflinger et al., 2017) and loss of control over eating (^*^Ivezaj et al., 2021). Moreover, NES patients also tended to indulge in higher levels of binge eating as compared to a control group (^*^Dalle Grave et al., 2013; ^*^Latzer et al., 2020a). On selected subscales of the EDI-2 (Garner et al., 1983)—drive for thinness, interceptive awareness and impulse regulation—higher scores were observed for patients with NES as compared to the control group (^*^Vinai et al., 2015b). Moreover, it was found that individuals with more severe NES symptoms received higher scores on the EDE (Fairburn and Beglin, 1994) subscales of dissatisfaction and over-evaluation (^*^Ivezaj et al., 2021).

Further, NES patients have been found to have elevated levels of internalized psychopathological symptoms. Studies that considered mood as an outcome (*n* = 4; ^*^Cleator et al., 2014; ^*^Dorflinger et al., 2017; ^*^Latzer et al., 2020a; ^*^Ivezaj et al., 2021) revealed that NES was linked to higher depression levels (measured through the BDI; Beck et al., 1961) as compared to controls and individuals with subthreshold NES symptoms. ^*^Dorflinger et al.'s (2017) study focused on veterans with NES, hence, demonstrating a comorbidity between NES to Post-traumatic Stress Disorder as well. Conversely, ^*^Latzer et al.'s (2020b) study found no evidence for a relationship between NES and childhood trauma or self-esteem levels. Self-reported anxiety was also found to be elevated in individuals with disordered night eating (^*^Cleator et al., 2014). However, there were no between group differences in individuals with full-threshold and sub-threshold NES (^*^Cleator et al., 2014).

Since NES is a nocturnal ED, it is often associated with poorer sleep-related outcomes. For example, night eaters tend to have poorer sleep quality (^*^Cleator et al., 2013; ^*^Ivezaj et al., 2021) and higher levels of insomnia (^*^Dorflinger et al., 2017). ^*^Loddo et al.'s (2019) study also found differences in sleep features in NES subgroups of evening hyperphagia (EH; those consuming ≥ 25% of total daily calories after the evening meal and may/may not indulge in nocturnal ingestion) and nocturnal ingestion (NI; those with only episodes of eating food at night). Sleep-related features such as eating latency (i.e., time gap between awakening from sleep and the eating episode), sleep latency after eating offset (i.e., time gap between the eating episode and returning to sleep), and the total eating episode duration, were found to be higher for the EH subgroup than the NI subgroup. Conversely, sleep duration, sleep efficiency, and rapid eye movement sleep remained unaffected for night eaters in some other studies (^*^Cleator et al., 2014; ^*^Loddo et al., 2019).

Only a few studies assessed the relationship between NES and personality traits. Two studies (^*^Dalle Grave et al., 2013; ^*^Vinai et al., 2015a) found significant differences on two of the TCI subscales (Cloninger et al., 1994)—harm avoidance and self-directedness—between the NES and the control group (^*^Dalle Grave et al., 2013; ^*^Vinai et al., 2015a). The NES group received higher scores on the harm avoidance subscale and lower scores on the self-directedness scales of the TCI (^*^Dalle Grave et al., 2013; ^*^Vinai et al., 2015a). With respect to impulsivity, NES sufferers were found to score higher on Barratt's Impulsiveness Scale (Barratt, 1965) than the control group (^*^Uncu et al., 2020). Finally, a study by ^*^Vinai et al. (2015b) revealed that the NES group displayed higher scores on alexithymia (i.e., the personality trait linked to the inability to identify and describe one's own emotions) than controls.

### Treatment Outcomes

This review included a total of four studies (^*^McCune and Lundgren, 2015; ^*^Vander Wal et al., 2015; ^*^Ferreira Pinto et al., 2017; ^*^Nasirzadeh et al., 2018) that explored the treatment modalities available for NES patients. The few available treatment options for NES currently include undergoing bariatric surgery (^*^Ferreira Pinto et al., 2017; ^*^Nasirzadeh et al., 2018), bright light therapy (BLT; ^*^McCune and Lundgren, 2015) and progressive muscle relaxation (PMR; ^*^Vander Wal et al., 2015) therapy.

The purpose of bariatric surgery is not to reduce NES related symptoms as the surgery is offered to individuals suffering from obesity to manage their health outcomes due to their increased weight. However, the surgery has been found to reduce NES-related dysfunction in patients' post-operative functioning (^*^Ferreira Pinto et al., 2017; ^*^Nasirzadeh et al., 2018). However, it needs to be outlined that the treatment's effects seem to be short-lived, as night eating scores were found to increase (*p* = 0.01; ^*^Nasirzadeh et al., 2018) between the second and the third post-operative years.

^*^McCune and Lundgren (2015) have successfully developed and provided evidence for the effectiveness of bright light therapy in improving outcomes for patients with NES. They found that exposing the NES participants with 10,000 lux lights for 14 consecutive morning sessions lasting 60 minutes each was found to reduce their NES symptoms, depressed mood, and insomnia (^*^McCune and Lundgren, 2015).

Progressive muscle relaxation (PMR) therapy is another treatment option proposed by ^*^Vander Wal et al. (2015). Their research demonstrated a 30.54% reduction in food intake after the evening meal in the group who practiced PMR, while also reducing depressive and anxiety symptoms (^*^Vander Wal et al., 2015).

Unfortunately, none of these studies had overlapping measures and/or data to provide support for the efficacy and effectiveness for these treatment options.

### Night Eating Syndrome and Binge Eating Disorder

One of the research aims of this review was to compare NES with BED and to throw some light onto the debate over the overlap of these two disorders. Three studies (^*^Roer et al., 2014; ^*^Baldofski et al., 2015; ^*^Zickgraf et al., 2019; summarized in Table 2) found evidence for differences between NES and BED on multiple outcomes. For instance, ^*^Baldofski et al. (2015) found that patients with BED had a greater number of objective and subjective binge eating episodes compared to NES patients. The same study also revealed that BED patients had a higher number of global ED symptoms, including eating concerns, weight and shape related concerns (^*^Baldofski et al., 2015). BED patients also engaged in emotional eating more frequently than NES patients as a response to negative emotions, or negative situations, or a combination of both (^*^Roer et al., 2014). A key difference between the two disorders was also demonstrated by ^*^Zickgraf et al.'s (2019) study, where the authors found that depressive symptoms fully mediated the relationship between BED and food insecurity, but only partially mediated the relationship between NES and food insecurity.

### Quality Appraisal

The results of the quality appraisal of all studies are represented in Table 3. Discrepancies between the coders were <12%. They were later resolved by discussing them with IK Almost all studies can be accredited for using appropriate statistical tests and valid outcome measures in their evaluation of NES and other variables. As 77% of the studies employed clinical convenience samples, criteria 7 was marked as zero for 17 out of the 22 studies. Criteria 5 was marked as not applicable (N/A) for most of the studies because of their cross-sectional design without a follow-up component. However, unfortunately, not all studies (e.g., ^*^Ferreira Pinto et al., 2017; ^*^Loddo et al., 2019; ^*^Kara et al., 2020) were found to control for confounding variables and were marked as zero for criteria 12 (see Table 3).

**Table 3:** Quality appraisal of the 22 selected studies using the 11 criteria adapted from Rozenblat et al. (2017).

**References**	**C1**	**C2**	**C3**	**C4**	**C5**	**C6**	**C7**	**C9**	**C10**	**C11**	**C12**
Baldofski et al. (2015)	1	1	1	1	0	1	0	1	1	1	1
Cleator et al. (2013)	1	1	1	1	N/A	1	0	1	1	N/A	1
Cleator et al. (2014)	1	1	1	1	1	1	0	1	1	1	1
Dalle Grave et al. (2013)	1	1	1	1	0	1	1	1	1	0	1
Dorflinger et al. (2017)	1	1	1	1	N/A	1	0	1	1	N/A	1
Ferreira Pinto et al. (2017)	1	1	1	1	N/A	1	0	1	1	N/A	0
Ivezaj et al. (2021)	1	1	1	1	N/A	1	0	1	1	N/A	1
Kara et al. (2020)	1	1	1	1	N/A	1	0	1	1	N/A	0
Latzer et al. (2020a)	1	1	1	1	N/A	1	1	1	1	1	1
Latzer et al. (2020b)	1	1	1	1	N/A	1	0	1	1	1	1
Loddo et al. (2019)	1	1	1	1	N/A	1	0	1	X	N/A	0
McCune and Lundgren (2015)	1	1	1	1	1	1	0	1	1	N/A	0
Nasirzadeh et al. (2018)	1	1	1	1	1	1	0	1	1	N/A	1
Roer et al. (2014)	1	1	1	1	N/A	1	0	1	1	1	1
Royal et al. (2015)	1	1	1	1	N/A	1	0	1	1	1	0
Tu et al. (2019)	1	1	1	1	N/A	1	1	1	1	0	1
Uncu et al. (2020)	1	1	1	1	N/A	1	0	1	1	X	0
Vander Wal et al. (2015)	1	1	1	1	0	1	1	1	1	N/A	X
Vinai et al. (2015a)	1	1	1	1	N/A	1	0	1	X	0	1
Vinai et al. (2015b)	1	1	1	1	N/A	1	1	1	1	1	1
Zengin-Eroglu et al. (2018)	1	1	1	1	N/A	1	0	1	X	N/A	1
Zickgraf et al. (2019)	1	1	1	1	N/A	1	0	1	1	N/A	1

*1 = Criteria met; 0 = Criteria not met; X = Unable to Determine; N/A = Criteria not Applicable.*

*Criteria Description: 1) Clear description of the hypothesis/aim/objectives; 2) Clear description of main outcomes in introduction/method; 3) Participant characteristics clearly described (as appropriate for ED research); 4) Clear description of main findings; 5) Characteristics of participants lost to follow-up described; 6) Exact probability values reported (or confidence intervals included); 7) Participants representative of population (including clinical, but not convenience samples); 9) Appropriate statistical tests used; 10) Main outcome measures valid and reliable; 11) Participants in different groups (if case–control study) recruited contemporaneously; and 12) Adequate adjustment for (potential) confounding variables (e.g., BMI)*.

## Discussion

To the authors' best knowledge, this is the first systematic review of the literature on NES in ED, obese or bariatric surgery clinical populations post the publication of DSM-5 (American Psychiatric Association, 2013); wherein NES has been recategorized as an OSFED and described using Allison et al.'s (2010) proposed diagnostic criteria. Very limited information about sociodemographic features (such as, age, gender, race, etc.), clinical features, comorbid conditions, including sleep disorders, and treatment options for NES are currently known.

Therefore, this review focused on collating these limited resources on NES. Another aim of the review was to find evidence of the differences or similarities between NES and BED in the chosen subpopulation, since the alleged overlap between the two disorders may discredit the validity of NES as an independent ED (Stunkard et al., 1996). Lastly, this review aimed to critically appraise the quality of studies available in the NES literature to draw appropriate conclusions from them.

### Night Eating Syndrome— Sociodemographic Features and Prevalence

The findings of this review suggest that NES is a relatively low prevalence disorder with rates ranging from 3 to 15% in most studies (^*^Cleator et al., 2013; ^*^Dalle Grave et al., 2013; ^*^Baldofski et al., 2015; ^*^Dorflinger et al., 2017; ^*^Ivezaj et al., 2021). However, these prevalence rates are larger than the lifetime prevalence rates of the other officially diagnosed EDs—Anorexia Nervosa (0.80%), Bulimia Nervosa (0.28%) and BED (0.85%; Udo and Grilo, 2018). Moreover, multiple studies from this review found that NES occurred frequently in obese (i.e., up to 24.62%; ^*^Kara et al., 2020; ^*^Uncu et al., 2020) and bariatric surgery samples (i.e., up to 20%; ^*^Zengin-Eroglu et al., 2018; ^*^Zickgraf et al., 2019).

Although there are some discrepancies, a large proportion of studies (e.g., ^*^Dalle Grave et al., 2013; ^*^Cleator et al., 2014; ^*^Dorflinger et al., 2017) have reached a consensus that sociodemographic factors (e.g., age, gender, education, income level) are not related to a NES diagnosis. Though our review found that individuals with NES tended to have a lower employment rate, it is likely that this may not be a causal factor but rather a consequence of suffering from this syndrome (^*^Cleator et al., 2014; ^*^Latzer et al., 2020a).

It is also unclear if the BMI of an individual is predictive of a NES diagnosis, as contradictory findings have been observed. A few studies have indicated that NES patients tend to have a higher BMI (e.g., ^*^Vinai et al., 2015a; ^*^Latzer et al., 2020b), however other studies (e.g., ^*^Zengin-Eroglu et al., 2018; ^*^Ivezaj et al., 2021) did not reveal such an association. The cautious conclusion that a higher BMI may be a risk factor for NES should therefore be made, as a higher BMI could simply be a consequence of the night eating behaviors.

### Night Eating Syndrome and Eating Disorder Symptoms, General Psychopathology, and Personality

The current review also attempted to shed light on the relationship between NES and related comorbid psychological factors, such as ED symptoms, general psychopathology and personality. The results of the present review identified patterns of psychopathology that are commonly present in individuals with NES. Accordingly, the review found that NES was often comorbid with binge eating behaviors (^*^Dalle Grave et al., 2013; ^*^Dorflinger et al., 2017; ^*^Latzer et al., 2020b), bulimic eating (^*^Vinai et al., 2015a), loss of control eating (even though atypical; ^*^Royal et al., 2015), and insomnia (^*^Dorflinger et al., 2017)—most of which are indicative of the severity of eating pathology in NES patients. The results from the current review further suggest that such presentation may be coupled with psychological effects such as, an increase in depression (^*^Cleator et al., 2014; ^*^Dorflinger et al., 2017; ^*^Latzer et al., 2020a), anxiety (^*^Vander Wal et al., 2015), impulsivity (^*^Uncu et al., 2020), and alexithymia (^*^Vinai et al., 2015b), all of which have been found to have an overall negative effect on the quality of life of an individual with NES (^*^Royal et al., 2015).

In terms of personality, the current review found that the harm avoidance and self-directedness subscales of the TCI (Cloninger et al., 1994) measure were associated with NES symptoms (^*^Dalle Grave et al., 2013; ^*^Vinai et al., 2015a). The TCI harm avoidance scale measures the excessive worrying and pessimism present in NES patients, while the self-directedness scale assesses their ability to regulate and adapt their behavior according to the demands of the situation (^*^Dalle Grave et al., 2013). It is important for clinicians to identify individuals at risk of developing NES (possibly using personality trait cues) to match patients appropriately to any of the treatment programs that are currently being suggested to be effective for NES, such as the employment of bright light therapy (BLT; ^*^McCune and Lundgren, 2015) or progressive muscle relaxation (PMR) therapy (^*^Vander Wal et al., 2015).

### Night Eating Syndrome and Binge Eating Disorder

This review also aimed to find evidence of differences or similarities between NES and BED in the chosen subpopulation, since such insights may speak to the validity of considering NES as an independent ED. Although, comorbidity between BED and NES diagnoses were present in up to 51.7% of patients (^*^Tu et al., 2019), none of the studies included in this review elaborated on the similarities between BED and NES (as outlined by Allison et al., 2007). However, a few studies found evidence for the differences between the two disorders in terms of ED and general psychopathology (e.g., ^*^Baldofski et al., 2015; ^*^Dalle Grave et al., 2013). Specifically, compared to NES patients, patients with BED exhibit a higher rate of ED related pathology (e.g., higher objective binging episodes, higher levels of eating concerns and overvaluation of weight and shape; ^*^Baldofski et al., 2015). These findings are consistent with Fairburn et al.'s (2003) transdiagnostic model, which suggests that overvaluation of weight and shape are the cardinal features of EDs. An important finding differentiating NES and BED (despite them both being comorbid with depression) is that depressive symptoms have been found to fully mediate the relationship between BED and food insecurity but were found to mediate this relationship only partially for NES (^*^Zickgraf et al., 2019).

The studies assessing NES and BED also showed that the two disorders differed on how they engaged in emotional eating: BED patients tended to eat emotionally more than NES patients, as a response to both negative emotions, situations, and loss of control over eating (^*^Roer et al., 2014). Such findings provided support for the hypothesis that, although, the observable behaviors between BED and NES may overlap, the two conditions are likely to have two distinct underlying motivations to engage in the disordered eating behaviors. Particularly, their motivations may differ because of higher levels of emotional and loss of control eating in BED patients, as compared to patients with NES (Stunkard and Allison, 2003). NES patients tend to “snack” at abnormal times (i.e., after the evening meal), however, they demonstrate a greater control over their abnormal eating behaviors than BED patients (Stunkard and Allison, 2003). Perhaps the reason why night eaters indulge in snacking at night lies in the shame felt by them for giving into the temptation of binging and the fear of being caught doing so during the daytime hours. However, there is a lack of exploration of the motivations behind night eating behaviors in NES patients, which is a key question that researchers should explore in future studies.

### Night Eating Syndrome and Sleep Disorder

Since there is an overlap in the symptom presentation between NES and Sleep-Related Eating Disorder (e.g., initiating and maintaining sleep, poor sleep quality and decreased total sleep time; ^*^Cleator et al., 2013; ^*^Loddo et al., 2019), this review also obtained evidence that sheds further light on its classification as an ED. Even with the efforts to synthesize the literature to settle this question, the results from the current findings are somewhat conflicting. From observations of abnormal eating patterns, such as multiple incidents of evening hyperphagia and nocturnal ingestion per week, ^*^Tu et al. (2019) found evidence in favor of NES being classified as an ED (cf. Latzer and Tzischinsky, 2012b). In contrast, the opinion by Schenck and Mahowald's (1994) that NES might be a sleep disorder, was supported in the current review by ^*^Dorflinger et al.'s (2017) paper, which found that most NES patients presented with insomnia. They concluded this because in their study, the abnormal eating patterns disrupted the NES participant's sleep. However, it should be noted that this study was conducted on veterans with obesity. Therefore, the study by ^*^Dorflinger et al. (2017) may not be generalisable to non-veteran populations. Overall, even though there is an overlap in the pathological features of NES and SRED, these conclusions are inconsistent and based on a very small number of studies. Therefore, future research that directly investigates the differences and similarities between NES and SRED is needed. Only with additional data is it possible to settle the question of whether NES might be best placed into the Feeding and Eating Disorder or Sleep Disorder category of DSM-5 (American Psychiatric Association, 2013).

### Quality Appraisal of the Literature

Another issue in this review is the quality of literature available on NES. Although the research papers included are of high quality and have been published in peer-reviewed journals (see Table 3), most of them have cross-sectional research designs which employed questionnaires and surveys (e.g., ^*^Cleator et al., 2013). Very few studies used an experimental design (e.g., ^*^McCune and Lundgren, 2015), though some studies (e.g., ^*^Cleator et al., 2014; ^*^Loddo et al., 2019) did make comparisons between sub-groups of NES patients (i.e., EH and NI subgroups, or full- and sub-threshold NES groups). Moreover, hardly any study focused exclusively on NES individuals, but most studies included NES patients that presented with a comorbidity of either obesity (e.g., ^*^Dalle Grave et al., 2013) or another ED subtype (e.g., ^*^Tu et al., 2019). Therefore, the information extracted from these studies about NES may be impacted by these comorbid diagnoses. Notwithstanding this limitation, it must also be acknowledged that in clinical populations, NES often is presented with these comorbidities; therefore, these samples do in fact reflect the true population under consideration.

### Theoretical and Clinical Implications

This review offers important theoretical and clinical implications. We have found further support for the notion that NES is highly prevalent in those suffering from obesity and BED, and those undergoing bariatric surgery. However, in most instances, individuals with NES are unaware of their conditions, since health professionals are overshadowed by comorbid conditions. Thus, individuals with NES are often unable to receive appropriate mental health care, which is further worsened by the dearth of research on treatment modalities for NES. Therefore, health professionals should be made aware of NES and its high prevalence in the relevant populations. Further, health professionals should be trained to screen patients for NES using validated screening tools (such as the Night Eating Questionnaire; Allison et al., 2008). They should consider a wider range of symptoms a patient might present with to guide their clinical decision-making process. Furthermore, our review suggests that NES and BED should be considered as two distinctive EDs despite the high rate of comorbidity among these two disorders. Therefore, mental health professionals should include the underlying characteristics of both NES and BED patients into their formulation, to provide the most individualized and appropriate care for these patients. Finally, based on the current literature (e.g., ^*^Dorflinger et al., 2017; ^*^Tu et al., 2019), it appears that NES shares similarities with both SRED and EDs. This implies that interventions for NES may need to address disordered eating behaviors and emotion regulation as well as poor sleep habits. Overall, given the complexity of NES, treatment should be adjusted on an individual basis, combining psychoeducation on diet and nutrition, and psychotherapy to yield results.

### Strengths, Limitations, and Future Direction

The current review has a few limitations that need to be acknowledged. First, given that the DSM-5 (American Psychiatric Association, 2013) is governed by Western thinking and most studies included in our review (e.g., ^*^Roer et al., 2014; ^*^Baldofski et al., 2015; ^*^Zickgraf et al., 2019) were conducted in Western countries, the results may not be generalisable to non-Western countries. Social and cultural backgrounds are considered significant contributing factors in the development of EDs (Miller and Pumariega, 2001). Given such factors would significantly differ in non-Western countries, future research should focus on identifying the clinical presentation of NES among individuals from a range of different cultural and ethnic backgrounds.

Second, most studies (e.g., ^*^Ferreira Pinto et al., 2017; ^*^Ivezaj et al., 2021) did not focus on finding evidence to solve the debate around how NES should be classified—(a) whether it should be categorized as a sleep disorder or ED in the DSM, and (b) whether it should be classified as an independent ED. Therefore, future studies would benefit from finding answers to these crucial issues. Furthermore, all studies included in this review were cross-sectional studies, thus causality and direction of the relationship of the symptoms and comorbidities assessed could not be determined. Therefore, the conduction of replication studies using different data collection methods, including longitudinal and more rigorous experimental designs, is deemed to be necessary.

Third, most studies included in the review examined mainly female (ranging from 65 to 100% of the samples across studies) NES patients. However, given that NES is found to be equally common in males and females, and that differences in clinical presentation between these two genders have been suggested (Murray et al., 2017), future research using a gender-diverse sample is needed to reach generalisable findings.

This review also had several strengths. First, there was a good variety of sample types in the studies reviewed, reporting several kinds of clinical presentations, including patients with obesity, various ED subtypes, and those undergoing bariatric surgery. Second, the studies included in this review went through a rigorous selection process from the authors to reduce any potential biases. Relatedly, this review provided insight into the quality of studies included in the review, which allowed us to make methodological recommendations for future studies.

## Conclusion

The current review provided insights into the current state of the NES literature in the BED, obesity, and bariatric surgery clinical populations. Several recent studies (e.g., ^*^Cleator et al., 2013; ^*^Loddo et al., 2019) focused primarily on NES patients, however, the literature on this OSFED subtype is still limited. Overall, this systematic review was able to lay out key sociodemographic features of NES, with significant data about the prevalence of NES in ED, obese and bariatric surgery populations. It has also consolidated information about ED pathology, eating patterns, emotional eating and internalizing disorders in NES and BED patients. Significant evidence from high quality studies distinguishing NES from BED has also been found. It is hoped that the strengthened evidence for NES obtained through the current review in the selected clinical populations, will inform treatment options for NES patients with the aim to improve their quality of life.

## Data Availability Statement

The datasets presented in this study can be found in online repositories. The names of the repository/repositories and accession number(s) can be found in the article/Supplementary Material.

## Author Contributions

JK, AD, and IK drafted the manuscript and JK and IK conceptualized the aims for the review. JK, ZA, and JG conducted the searches and undertook the quality appraisal. All authors provided feedback on different versions of the manuscripts, read and approved the final manuscript, and are accountable for all aspects of the work in ensuring that questions related to the accuracy of any part of the work are appropriately investigated.

## Conflict of Interest

The authors declare that the research was conducted in the absence of any commercial or financial relationships that could be construed as a potential conflict of interest.

## Publisher's Note

All claims expressed in this article are solely those of the authors and do not necessarily represent those of their affiliated organizations, or those of the publisher, the editors and the reviewers. Any product that may be evaluated in this article, or claim that may be made by its manufacturer, is not guaranteed or endorsed by the publisher.
