# Supplementary material for: Night Eating Syndrome in Patients With Obesity and Binge Eating Disorder: A Systematic Review
Source: Front Psychol. 2022 Jan 5;12:766827. doi: 10.3389/fpsyg.2021.766827 (PMC8766715; doi:10.3389/fpsyg.2021.766827)
Supplement: Supplementary file 1 [file Table_1.DOCX]

**Supplementary File 1:**

**Search Syntax**

**Databases used:** MEDLINE, Embase, PubMed

**Filters applied:**

- Through database search:
  - Humans
  - English language

**Part A:**

**Search Terms:**

"night eating disorder" OR "night eating" OR "late night eating" OR "night eating syndrome"

**MEDLINE:**

| # | Search | Results |
| --- | --- | --- |
| 1 | ("night eating disorder" or "night eating" or "late night eating" or "night eating syndrome").mp. [mp=title, abstract, original title, name of substance word, subject heading word, floating sub-heading word, keyword heading word, organism supplementary concept word, protocol supplementary concept word, rare disease supplementary concept word, unique identifier, synonyms] | 408 |
| 2 | limit 1 to (english language and humans) | 307 |

**Embase:**

| # | Search | Results |
| --- | --- | --- |
| 1 | ("night eating disorder" or "night eating" or "late night eating" or "night eating syndrome").mp. [mp=title, abstract, heading word, drug trade name, original title, device manufacturer, drug manufacturer, device trade name, keyword, floating subheading word, candidate term word] | 641 |
| 2 | limit 1 to (human and english language) | 552 |

**PubMed:**

| # | Search | Results |
| --- | --- | --- |
| 1 | ("night eating disorder" or "night eating" or "late night eating" or "night eating syndrome") | 593 |
| 2 | limit 1 to (human and english language) | 438 |

***Total:*** 1297

***After removing duplicates using EndNote:*** 722

These were imported to Covidence.

After screening, 35 duplicates were removed.

**687 papers** need to be reviewed.

**Part B:**

**Search Terms:**

"night eating disorder" OR "night eating" OR "late night eating" OR "night eating syndrome"

AND

"binge eating disorder" OR "binge eating" OR "binge" OR "binging"

**Filters applied:**

- Through database search:
  - Human
  - English language

**MEDLINE:**

| # | Search | Results |
| --- | --- | --- |
| 1 | ("night eating disorder" or "night eating" or "late night eating" or "night eating syndrome").mp. [mp=title, abstract, original title, name of substance word, subject heading word, floating sub-heading word, keyword heading word, organism supplementary concept word, protocol supplementary concept word, rare disease supplementary concept word, unique identifier, synonyms] | 408 |
| 2 | limit 1 to (english language and humans) | 307 |
| 3 | ("binge eating disorder" or "binge eating" or "binge" or "binging").mp. [mp=title, abstract, original title, name of substance word, subject heading word, floating sub-heading word, keyword heading word, organism supplementary concept word, protocol supplementary concept word, rare disease supplementary concept word, unique identifier, synonyms] | 15689 |
| 4 | limit 3 to (english language and humans) | 11425 |
| 5 | 2 and 4 | 105 |

**Embase:**

| # | Search | Results |
| --- | --- | --- |
| 1 | ("night eating disorder" or "night eating" or "late night eating" or "night eating syndrome").mp. [mp=title, abstract, heading word, drug trade name, original title, device manufacturer, drug manufacturer, device trade name, keyword, floating subheading word, candidate term word] | 641 |
| 2 | limit 1 to (human and english language) | 552 |
| 3 | ("binge eating disorder" or "binge eating" or "binge" or "binging").mp. [mp=title, abstract, heading word, drug trade name, original title, device manufacturer, drug manufacturer, device trade name, keyword, floating subheading word, candidate term word] | 25015 |
| 4 | limit 3 to (human and english language) | 19926 |
| 5 | 2 and 4 | 356 |

**PubMed:**

| # | Search | Results |
| --- | --- | --- |
| 1 | ("night eating disorder" or "night eating" or "late night eating" or "night eating syndrome") | 593 |
| 2 | limit 1 to (human and english language) | 438 |
| 3 | ("binge eating disorder" or "binge eating" or "binge" or "binging") | 15728 |
| 4 | limit 3 to (human and english language) | 11451 |
| 5 | ("night eating disorder" or "night eating" or "late night eating" or "night eating syndrome") AND ("binge eating disorder" or "binge eating" or "binge" or "binging") | 148 |
| 6 | limit 5 to (human and english language) | 116 |

- ***Total:*** 577
- ***After removing duplicates using EndNote:*** 362
- Finding duplicates when compared to the first search. Manually removed papers that have been screened already.
- Papers remaining for screening: ***Zero***
